# Towards continuous-to-continuous 3D imaging in the real world

**DOI:** 10.1088/1361-6560/ab3fb5

**Published:** 2019-09-18

**Authors:** L Caucci, Z Liu, A K Jha, H Han, L R Furenlid, H H Barrett

**Affiliations:** 1 Department of Medical Imaging, University of Arizona, Tucson, AZ 85724, United States of America; 2 College of Optical Sciences, University of Arizona, Tucson, AZ 85719, United States of America; 3 Washington University School of Medicine, St. Louis, MO 63110, United States of America; 4 Molecular Medicine Division, Translational Genomics Research Institute, Phoenix, AZ 85004, United States of America; 5 Author to whom any correspondence should be addressed.

**Keywords:** emission tomography, SPECT, reconstruction, MLEM, graphics processing units, GPU

## Abstract

Imaging systems are often modeled as continuous-to-discrete mappings that map the object (i.e. a function of continuous variables such as space, time, energy, wavelength, etc) to a finite set of measurements. When it comes to reconstruction, some discretized version of the object is almost always assumed, leading to a discrete-to-discrete representation of the imaging system. In this paper, we discuss a method for single-photon emission computed tomography (SPECT) imaging that avoids discrete representations of the object or the imaging system, thus allowing reconstruction on an arbitrarily fine set of points.

## 1. Introduction

Mathematical tomography centers around integral transforms in which both the object and the image are treated as functions of continuous variables; we refer to an integral transform as a continuous-to-continuous (CC) operator.

The ubiquitous practice of representing real-life objects (such as organs, bones, tissues, etc in the case of medical imaging) with a finite set of intensities over a 2D or 3D grid of pixels or voxels often leads to inaccuracies when the object itself presents many features that cannot be represented using a grid of pixels or voxels. Similarly, sets of discrete data (such as projection images in the case of emission tomography) are typically used as the starting point to perform reconstruction. These discretizations of the object and the data produced by the imaging system are almost always assumed, leading to matrix representations or discrete-to-discrete (DD) operators.

In this paper, we discuss our approach to approximating CC operators for real single-photon emission computed tomography (SPECT) imaging systems in which we use measured calibration data from real detectors and real multi-pinhole imaging systems, thus potentially avoiding discrete representations of the object or the raw data. We use photon-processing detectors to collect data with no binning involved, and show how such data sets can be reconstructed using iterative maximum-likelihood (ML) estimation algorithms. Our approach does not introduce any error due to discretization of the measurement, and it allows reconstructions over an arbitrary sets of points, not necessarily arranged as a 3D uniform grid of voxels.

Prior art in list-mode reconstruction can be found in the work by Levkovitz *et al* (2001). In it, the authors derive an algorithm for direct reconstruction of list-mode data for positron emission tomography (PET) by starting from an expression of the maximum-likelihood expectation maximization algorithm for binned data, and then they consider the limit of no more than one count per bin. An alternative formulation, also for PET imaging, is provided in Parra and Barrett (1998). Similar to Barrett *et al* (1997) and Parra and Barrett (1998), our algorithm assumes that the list-mode data are positions of interaction as estimated (Moore *et al* 2007) from data collected with the gamma-ray cameras in the SPECT scanner. These estimates are allowed to vary over a continuous domain, and probability density functions are evaluated on-the-fly for each item of the list.

This paper is organized as follows. Section 2 presents the mathematical notation used in this paper; section 3 discusses photon-processing detectors and introduces a point process we use to mathematically represent list-mode data. In section 4 we discuss the CC imaging operator as a mapping from the object space to data space. This treatment allows us to derive an explicit expression for the kernel of this CC operator. In section 5 we present our reconstruction approach, which is based on the list-mode maximum-likelihood expectation-maximization (MLEM) algorithm. In section 6 we discuss reconstruction results with real data. Future work and conclusions are provided in sections 7 and 8, respectively.

This paper is an extended version of the conference proceeding (Caucci *et al* 2015a) presented by the authors at the *13th International Meeting on Fully Three-Dimensional Image Reconstruction in Radiology and Nuclear Medicine* (‘Fully 3D 2015’) held in Newport, Rhode Island, USA.

## 2. Mathematical formulation

In the absence of detector noise, imaging systems are often modeled as linear continuous-to-discrete (CD) operators H that map the object function *f* (***r***) to a set of numbers $\bar{g}$. In abstract form, this mapping is written as (Barrett and Myers 2004):

(1) $$\bar{g}=Hf,$$

in which **f** corresponds to the object function *f* (***r***) and belongs to the Hilbert space of square-integrable functions of the variable ***r***. In our treatment, we will consider object functions *f* (***r***) that depend only on the 3D continuous variable ***r***; more general cases of object functions depending on other variables are possible. We will further restrict our attention to SPECT imaging, and will assume that *f* (***r***) Δ***r*** Δ*t* is the mean number of gamma-ray photons emitted isotropically from a small volume Δ***r*** (centered around point ***r***) and during time interval Δ*t*. Hence, the units of *f* (***r***) are photons/(s · m^3^). Finally and unless otherwise stated, we will ignore changes in nuclear activity due to radioactive decay.

If we assume that $\bar{g}$ is a vector with *M* components denoted as ${\bar{g}}_{1},\ldots ,{\bar{g}}_{M}$, then the expression in (1) can be written in component form as

(2) $${\bar{g}}_{m}=\int_{S}h_{m}(r)f(r){\text{d}}^{3}r,$$

in which ∫_*S*_ … denotes integration over the support *S* of the object *f* (***r***), and the function *h*_*m*_(***r***) represents the system response at point ***r*** in the object and for the *m*th element of $\bar{g}$.

In the development of reconstruction algorithms, it is customary to characterize the imaging system with a system matrix *H*, so that a discrete representation ***f*** of the object function *f* (***r***) can be related to the noise-free data $\bar{g}$ according to the following equation (Barrett and Myers 2004):

(3) $$\bar{g}=Hf.$$

In the expression above, ***f*** is a vector with *N* components *f*_1_, …, *f*_*N*_ and *H* is an *M* × *N* matrix with components *h*_*m*,*n*_. In component form, the expression in (3) becomes

(4) $${\bar{g}}_{m}=\sum_{n=1}^{N}h_{m,n}f_{n}.$$

Because both ***f*** and $\bar{g}$ are vectors of finite size, this representation is often referred to as discrete-to-discrete (DD) representation (Barrett and Myers 2004).

One can relate the function *f* (***r***) to the discrete vector ***f***, and the kernel *h*_*m*_(***r***) of H to the elements *h*_*m*,*n*_ of the matrix *H* by first assuming that there exist functions *ϕ*_1_(***r***), …, *ϕ*_*N*_(***r***) so that

(5) $$f(r)=\sum_{n=1}^{N}f_{n}\phi_{n}(r),$$

in which *f*_1_, …, *f*_*N*_ are real numbers. Upon substitution, we obtain:

(6) $${\bar{g}}_{m}=\int_{S}h_{m}(r)f(r){\text{d}}^{3}r$$

(7) $$=\sum_{n=1}^{N}\left[\int_{S}h_{m}(r)\phi_{n}(r){\text{d}}^{3}r\right]f_{n},$$

which shows

(8) $$h_{m,n}=\int_{S}h_{m}(r)\phi_{n}(r){\text{d}}^{3}r.$$

If functions *ϕ*_1_(***r***), …, *ϕ*_*N*_(***r***) are orthonormal, the numbers *f*_1_, …, *f*_*N*_ are calculated from *f* (***r***) according to

(9) $$f_{n}=\int_{S}f(r)\phi_{n}(r){\text{d}}^{3}r.$$

It is worth noting that the condition in (5) is very restrictive. Real-world objects are often complicated and cannot, in general, be described as a finite sum as in (5). Hence, the formalism of (3) is fundamentally flawed and the usual way to circumvent this fallacy is to consider approximated versions of many of the expressions above.

In this work, we use a more accurate representation of the imaging system, which avoids introducing approximations. Mathematically, we consider a mapping L between the object **f** and a new function $\bar{u}(A)$:

(10) $$\bar{u}=Lf,$$

in which $\bar{u}$ belongs to a Hilbert space and corresponds to the function $\bar{u}(A)$ of the continuous variable ***A*** (to be discussed below). Because variable ***A*** is allowed to take on continuous values, the mapping above, characterized by the operator L, is a continuous-to-continuous (CC) mapping.

## 3. Photon-processing detectors

The CC systems discussed here do not collect data as sets of pixel counts. Instead, they make use of photon-processing detectors (Caucci *et al* 2013, Jha *et al* 2015, Ding *et al* 2017a, Caucci *et al* 2018) to collect data. We define a photon-processing detector as any detector that (Caucci *et al* 2015b):

- uses a gain mechanism and multiple sensors to obtain multiple measurements for a single absorbed photon;
- collects data from all sensors that respond to each event at full precision;
- uses the sensor data and maximum-likelihood estimation to estimate a vector of parameters (or ‘attributes’);
- stores all ML estimates of attributes in a list, at full precision.

For example, in the case of SPECT imaging with Anger cameras (Anger 1958, Peterson and Furenlid 2011), a photon-processing detector utilizes all available photomultiplier tube (PMT) signals that have been properly sampled and converted to digital numbers to estimate attributes (such as 2D or 3D position within the camera’s crystal, photon energy, and so on) of gamma-ray photons interacting with the camera. These maximum-likelihood estimates are not restricted to belong to finite sets of values (as in the case of DD systems), but they can take on any—potentially continuous—value.

The vector of attributes for the *j*th detected photon is denoted as ${\hat{A}}_{j}$, for *j* = 1, …, *J*, where *J* is the total number of photon detected during some time interval of length *T*. Vector ${\hat{A}}_{j}$ is a vector with *K* components, and it can consist of any attribute we can estimate from the detector outputs, such as the 2D or 3D position of interaction of the photon with the crystal, the energy deposited by the photon in the crystal, the time of arrival of the photon and the direction of propagation. We use the ‘hat’ symbol to mean that ${\hat{A}}_{j}$ is calculated by means of an estimation algorithm (such as an ML estimation algorithm (Dempster *et al* 1977)) from noisy detector outputs. The noise in the detector outputs is random, which makes ${\hat{A}}_{j}$ a random quantity as well.

**Figure 1.**
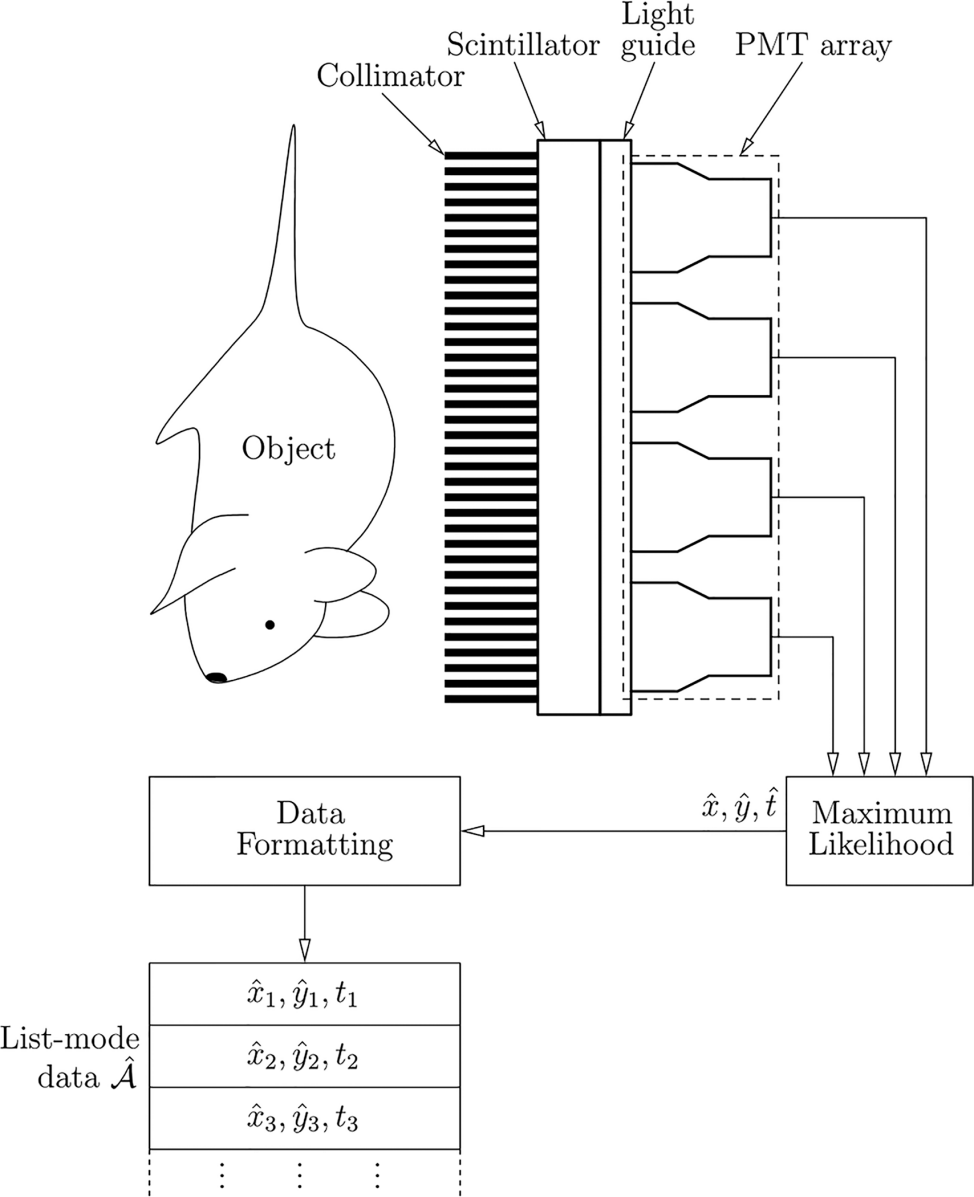
Example of a photon-processing imaging system (adapted from Caucci *et al* (2013)).

The list of attributes $\hat{A}=\left\{{\hat{A}}_{1},\ldots ,{\hat{A}}_{J}\right\}$ can mathematically be represented as a random point process (Lehovich 2005, Caucci and Barrett 2012, Caucci 2012, Jha 2013) ***u***:

(11) $$u(\hat{A})=\sum_{j=1}^{J}\delta \left(\hat{A}-{\hat{A}}_{j}\right),$$

where *δ*(…) is a *K*-dimensional Dirac delta function. Any imaging system that stores the list of attribute vectors $\hat{A}=\left\{{\hat{A}}_{1},\ldots ,{\hat{A}}_{J}\right\}$ (as opposed to pixel counts *g*_1_, …, *g*_*M*_) is said to be storing the data in list-mode format. A schematic representation of a SPECT photon-processing imaging system is shown in figure 1.

The formalism we defined here is not restricted to SPECT imaging with Anger cameras. In fact, applications of photon-processing detectors to imaging with charged particles (such as electrons or alpha and beta particles) as well as imaging in the visible range have recently appeared in the literature (Ding *et al* 2014a, 2014b, Barrett *et al* 2017, 2017a, 2017b, Ding *et al* 2017a, 2017b).

## 4. The imaging operator L

Though both H and L operators are linear and operate on the same object, the CC operator L is fundamentally different from the CD operator H. A CD operator necessarily has an infinite-dimensional null space (Barrett and Myers 2004); the system maps a vector in an infinite-dimensional Hilbert space to a finite set of numbers, so there is an infinite set of vectors ***f***_null_ that yield no data at all: $Hf_{\text{null}}=0$. Because real objects almost always have null components for any CD system, the object itself cannot be recovered from the discrete data, and even simple linear functionals such as integrals of the object over voxels cannot be estimated.

On the other hand, a CC operator such as L *may* have a null space, but for the setup of section 3 and if *K* ⩾ 3, the null space is not demanded by dimensionality considerations. Thus, it is possible that some—conceivably all—of the estimability problems inherent in the world of conventional digital imaging may disappear (Ding *et al* 2017b). Notice that replacing the operator H with L does not, in general, ensure that an inverse problem that was ill-posed (Natterer 1986, Bertero and Boccacci 1998) in the CD case becomes well-posed when a CC operator is used. As an example, although the Radon transform maps a continuous function to another continuous function, the problem its inverse solves is ill-posed.

To derive an explicit expression for the kernel of the CC operator L, we begin by considering the probability density function for attribute vector $\hat{A}$ for a given object ***f***; we will denote such a quantity as $\text{pr}(\hat{A}|f)$, in which the vertical line ‘\vert’ denotes conditioning with respect to a known quantity. In principle, one could use the Boltzmann transport equation to relate the source distribution ***f*** to the spectral photon radiance (Barrett and Myers 2004) at the detector’s face, and then express $\text{pr}(\hat{A}|f)$ as a blurred version of the properly scaled and marginalized spectral photon radiance (Caucci *et al* 2015b), with the blurring accounting for the inaccuracies in estimating $\hat{A}$ from the sensor data. In our treatment, however, we follow a derivation that explicitly takes into account the construction of the random point process ***u*** in (11) from list-mode data $\hat{A}$. By the properties of conditional probability, we have (Caucci and Barrett 2012):

(12) $$\text{pr}(\hat{A}|f)=\int_{S}\text{pr}(\hat{A}|r)\text{ pr}(r|f){\text{d}}^{3}r,$$

in which $\text{pr}(\hat{A}|r,c)$ is the probability density function for measuring $\hat{A}$ under the assumption that a gamma-ray photon was emitted from point ***r*** ∈ *S*. The probability density function pr(***r*** | ***f***) is given by

(13) $$\text{pr}(r|f)=\frac{s(r)f(r)}{\int_{S}s\left(r^{\prime }\right)f\left(r^{\prime }\right){\text{d}}^{3}r^{\prime }},$$

in which *s*(***r***) is the system’s sensitivity function, which is defined here as the probability for a photon emitted at point ***r*** to produce any signal. In other words:

(14) $$s(r)=\int_{\infty }\text{pr}(\hat{A}|r){\text{d}}^{K}\hat{A}.$$

Notice that pr(***r*** | ***f***) as defined above does not describe the probability density function for the emission point for an object ***f***. In fact, pr(***r*** | ***f***) is the probability density function for an emission from point ***r*** and an object ***f*** to produce a signal in the detector. A photon emitted from point ***r*** can just be blocked by the pinhole or, if it does not, it can go through the scintillator in the camera and produce no signal. The probability density function pr(***r*** | ***f***) as defined in (13) not only takes into account the distribution of the emission points (defined by ***f***) but also the probability of the emitted gamma-ray photon to produce a signal (defined by the system’s geometry).

In principle, *s*(***r***) can be calculated (either analytically or using numerical methods, such as Monte Carlo estimation (Fishman 1996)), at any point from knowledge of the system geometry, or it can be estimated by moving a point-like source across the system’s field of view and then by taking *s*(***r***) = *αJ*_***r***_ in which *J*_***r***_ is the number of attribute vectors collected during a sufficiently long time interval and when the point-like source was located at point ***r***, and *α* is a constant that makes *s*(***r***) the probability for a photon emitted at point ***r*** to produce any signal.

The probability density function $\text{pr}(\hat{A}|r)$ can be further rewritten to explicitly take into account estimation of $\hat{A}$ from noisy signals (e.g. discretized PMT outputs produced by the camera in the case of SPECT) and propagation of light from point ***r*** ∈ *S* to the camera’s entrance face:

(15) $$\text{pr}(\hat{A}|r)=\int_{\infty }\text{pr}(\hat{A}|A)\text{ pr}(A|r){\text{d}}^{K}A.$$

In the expression above, ***A*** denotes a noise-free event (i.e. what we would obtain in the case of an ideal, noise-free detector) and $\text{pr}(\hat{A}|A)$ characterizes the properties of the detector. It can be shown (Abbey *et al* 1998, Caucci *et al* 2010) that if $\hat{A}$ is a maximum-likelihood estimate of ***A*** (as it was assumed in figure 1), then, under fairly general conditions, $\hat{A}$ asymptotically (i.e. as the number of photo-electrons in the PMTs gets larger and larger) follows Gaussian statistics with mean ***A*** and covariance matrix given by the inverse of the Fisher information matrix (Van Trees 1968, Mardia *et al* 1979) at ***A***.

For any given object ***f***, the average $\bar{u}$ of ***u*** defined in (11) is

(16) $$\bar{u}(\hat{A})=\sum_{J=0}^{\infty }\text{Pr}(J|f,T)\underset{J\text{ integrals}}{\underset{︸}{\int_{\infty }\cdots \int_{\infty }}}\left[\sum_{j=1}^{J}\delta \left(\hat{A}-{\hat{A}}_{j}\right)\right]\;\times \left[\prod_{j=1}^{J}\text{pr}\left({\hat{A}}_{j}|f\right)\right]{\text{d}}^{K}{\hat{A}}_{1}\ldots {\text{d}}^{K}{\hat{A}}_{J}\;=\sum_{J=0}^{\infty }J\text{ Pr}(J|f,T)\int_{\infty }\delta \left(\hat{A}-{\hat{A}}^{\prime }\right)\text{pr}\left({\hat{A}}^{\prime }|f\right){\text{d}}^{K}{\hat{A}}^{\prime }\;=\bar{J}(f,T)\text{ pr}(\hat{A}|f),$$

in which Pr(*J* | ***f***, *T*) is the probability of measuring exactly *J* attribute vectors while imaging the object ***f*** for time *T*, and $\bar{J}(f,T)$ is the average of *J* under the same circumstances. It turns out (Lehovich 2005, Caucci and Barrett 2012):

(17) $$\bar{J}(f,T)=T\int_{S}s\left(r^{\prime }\right)f\left(r^{\prime }\right){\text{d}}^{3}r^{\prime }.$$

Upon substitution in the last expression in (16), we obtain

(18) $$\bar{u}(\hat{A})=T\int_{S}\text{pr}(\hat{A}|r)s(r)f(r){\text{d}}^{3}r,$$

which shows that the kernel of L is:

(19) $$[L](\hat{A},r)=T\text{ pr}(\hat{A}|r)s(r).$$

## 5. Reconstruction with FastSPECT II

### 5.1. System description

The imaging system we used for our study, called FastSPECT II, was developed at the Center for Gamma-Ray Imaging, University of Arizona. FastSPECT II is a small-animal SPECT imager built with modular scintillation cameras (Milster 1987, Milster *et al* 1990) and list-mode data-acquisition electronics. The modular gamma-ray scintillation camera designed for FastSPECT II comprises a 5 mm thick NaI(Tl) scintillation crystal, a 15 mm thick quartz light guide, and a 3 × 3 array of 1.5 inch diameter end-on photomultiplier tubes. Each camera has an input face measuring about 120 × 120 mm^2^ (Furenlid *et al* 2004, Chen 2006).

**Figure 2.**
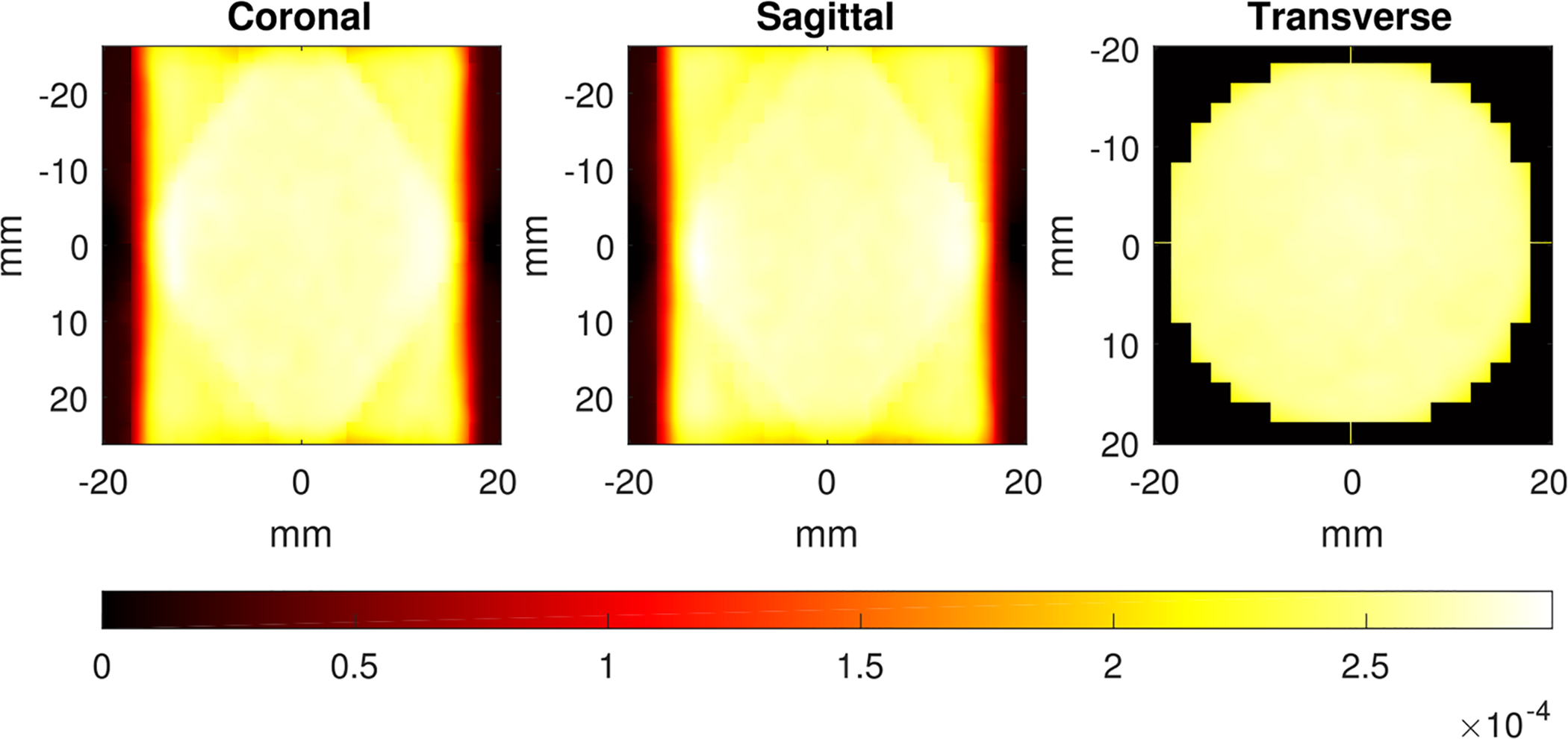
Plot of the sensitivity across three planes, showing almost uniform sensitivity over a large portion of the system’s field of view. The volume for which the sensitivity exceeds 5% its maximum value is about 57 cm^3^.

Each entry in the data list collected by a gamma-ray camera corresponds to a detected scintillation event and consists of a camera identifier, the nine signal values present in the 3 × 3 array of photomultipliers, and a time stamp. The cameras are stationary and arranged as two rings of eight on opposite sides of a pair of central plates (Furenlid *et al* 2004, Chen 2006). As shown in figure 2, FastSPECT II attains good sensitivity over a large volume—about 57 cm^3^—well suited for small-animal imaging. As reported in Chen (2006), the sensitivity of FastSPECT II at the center of the FOV is about 267 counts per second (cps) per each million gamma rays emitted per second. Hence, *s*(***r***_center_) ≈ 2.67 · 10^−4^.

### 5.2. Estimation of photon attributes

We used PMT signals *p*_1_, …, *p*_*I*_ (*I* = 9 for FastSPECT II) from each camera to perform ML estimation of photon attributes. The estimated attributes consisted of the 2D position of interaction between a gamma-ray photon and the camera’s entrance face. Hence, $\hat{A}=(\hat{X},\hat{Y})$. This ML estimate is defined as

(20) $$\hat{A}=\underset{A}{\text{arg max}}\text{ Pr}\left(p_{1},\ldots ,p_{I}|A\right),$$

in which Pr(*p*_1_, …, *p*_*I*_ | ***A***) is the probability of measuring PMT signals *p*_1_, …, *p*_*I*_ when a gamma-ray interaction occurs at point ***A*** = (*X*, *Y*) and the ‘arg max_***A***_’ notation in (20) denotes the value of ***A*** that maximizes Pr(*p*_1_, …, *p*_*I*_ | ***A***). The quantity *L*(***A***; *p*_1_, …, *p*_*I*_) = Pr(*p*_1_, …, *p*_*I*_ | ***A***), in which *p*_1_, …, *p*_*I*_ are assumed fixed is called ‘likelihood’ (Barrett and Myers 2004). Its logarithm, $l\left(A;p_{1},\ldots ,p_{I}\right)=\text{log}\left[L\left(A;p_{1},\ldots ,p_{I}\right)\right]$, is often referred to as ‘log-likelihood’.

Calculating the probability Pr(*p*_1_, …, *p*_*I*_ | ***A***) for a given ***A*** requires knowledge of the forward model and its statistical properties. A calibration procedure is often used to obtain information about the statistics of *p*_1_, …, *p*_*I*_ for points ***A*** on a regular grid (Peterson and Furenlid 2011, Chen 2006). Other approaches, including Monte Carlo estimation, have been considered (Hunter *et al* 2013). We will assume that *p*_1_, …, *p*_*I*_ conditioned on ***A*** are independent and follow Poisson statistics (Hunter 2007, Barrett *et al* 2009, Hunter *et al* 2009); under such hypotheses, we have:

(21) $$\text{Pr}\left(p_{1},\ldots ,p_{I}|A\right)=\prod_{i=1}^{I}\text{Pr}\left(p_{i}|A\right)$$

(22) $$=\prod_{i=1}^{I}\frac{{\left[{\bar{p}}_{i}(A)\right]}^{p_{i}}}{p_{i}!}e^{-{\bar{p}}_{i}(A)},$$

in which ${\bar{p}}_{i}(A)$ is often called mean detector response function (MDRF). For any *i* = 1, …, *I*, the MDRF ${\bar{p}}_{i}(A)$ is the mean of *p*_*i*_ under the assumption that a gamma-ray interaction has occurred at point ***A***. Although ${\bar{p}}_{i}(A)$ is usually estimated or measured for a finite set of points (typically arranged in a uniform pattern), in practice it is a smooth function of ***A*** and it is therefore possible to interpolate these samples so that ${\bar{p}}_{i}(A)$ can be evaluated for any ***A***.

After an ML estimate $\hat{A}$ is calculated as in (20), the likelihood $L\left(\hat{A};p_{1},\ldots ,p_{I}\right)$ is compared to a threshold $L_{0}(\hat{A})$. If $L\left(\hat{A};p_{1},\ldots ,p_{I})<L_{0}(\hat{A}\right)$, then estimate $\hat{A}$ is discarded. This technique, originally introduced in Milster *et al* (1990), is called ‘likelihood thresholding’ and it is used to ignore estimates $\hat{A}$ that poorly match the statistical model of (21). This is often due to scattering of gamma-ray photons and subsequent loss of photon energy (Chen 1997). Calculation of the position-dependent likelihood threshold $L_{0}(\hat{A})$ has been detailed in Chen (2006). Briefly, for any fixed ***A***_0_, raw PMT data *p*_1_, …, *p*_*I*_ collected in the calibration step are reprocessed to create histograms of log-likelihood $l\left(A_{0};p_{1},\ldots ,p_{I}\right)$. Sample mean and variance of the log-likelihood values are calculated. The algorithm iteratively discards values of log-likelihood that are outside two standard deviations around the sample mean, and recomputes the sample mean and variance until the mean converges to a fixed value with an error less than one decimal place (Chen 2006). A value $l_{0}\left(A_{0}\right)$ is set at four standard deviations below the sample mean. Finally, the likelihood threshold *L*_0_(***A***_0_) is set as $L_{0}\left(A_{0}\right)=\text{exp}\left[l_{0}\left(A_{0}\right)\right]$.

Reduced spatial resolution has been observed near the edges and corners of the crystal (Barrett *et al* 2009, Moore 2011) with a larger-than-expected number of position estimates clustering near the edges of the crystal; an example is shown in figure 4. This is likely due to a combination of effects, including non-monotonic signal variation as a function of position in these areas. Methods to cope with this problem have been proposed; they generally rely on the estimation of the total energy the gamma-ray photon has deposited in the crystal. In this work, we simply decided to ignore all estimates that are less than 4 mm away from any of the crystal edges.

Graphics processing unit (GPU) code was developed (Caucci and Furenlid 2015) to process PMT data and perform the 2D ML estimation of position of interaction in (20) for each detected gamma-ray photon. Our algorithm for ML estimation of position of interaction used an iterative contracting-grid approach (Furenlid *et al* 2005). We took full advantage of the texture mapping unit of the GPU devices to interpolate the PMT mean detector response function (MDRF) of each camera. The CUDA cubic B-spline interpolation library (Ruijters *et al* 2008) was used to perform on-the-fly interpolation of MDRF samples with low-degree spline functions (Cox 1972, Schumaker 1981). The main advantage of this contracting-grid approach is that 2D estimates can be performed with arbitrary precision by simply running more iterations of the contracting-grid search algorithm. Other customizations, such as contracting factor after each iterations and the size of the grid, are possible (Caucci and Furenlid 2015, Caucci *et al* 2018). Moreover, the same algorithm is well suited for the estimation of other photon attributes provided that the proper statistical model Pr(*p*_1_, …, *p*_*I*_ | ***A***) is available.

### 5.3. The list-mode MLEM reconstruction algorithm

A list-mode variant (Parra and Barrett 1998, Caucci 2012) of the maximum-likelihood expectation-maximization (MLEM) (Shepp and Vardi 1982) algorithm has been implemented on GPUs to perform reconstruction from list-mode data ${\hat{A}}^{\left(c\right)}=\left\{{\hat{A}}_{1}^{\left(c\right)},\ldots ,{\hat{A}}_{J_{c}}^{\left(c\right)}\right\},$, in which *c* = 1, …, *C* denotes the camera and *J*_*c*_ is the number of recorded events for camera *c*. In the formalism discussed in this paper, the list-mode MLEM algorithm is written as

(23) $${\hat{f}}_{n}^{\left(l+1\right)}=\frac{{\hat{f}}_{n}^{\left(l\right)}}{T}\sum_{c=1}^{C}\sum_{j=1}^{J_{c}}\frac{\text{pr}\left({\hat{A}}_{j}^{\left(c\right)}|r_{n},c\right)}{\sum_{n^{\prime }=1}^{N}\text{pr}\left({\hat{A}}_{j}^{\left(c\right)}|r_{n^{\prime }},c\right)s_{n^{\prime }}{\hat{f}}_{n^{\prime }}^{\left(l\right)}},$$

in which *T* is the exposure time, ${\hat{f}}_{n}^{\left(l\right)}$ is the estimate of *f*_*n*_ after l iterations and $\text{pr}\left({\hat{A}}^{\left(c\right)}|r,c\right)$ is the quantity calculated in (15) in which we have extended our notation to make it clear that attribute vector ${\hat{A}}^{\left(c\right)}$ corresponds to a recorded event in camera *c*. The iterative expression above requires an initial guess ${\hat{f}}_{n}^{\left(0\right)}$ for *n* = 1, …, *N*, which we will assume to be a non-negative constant; in other words, ${\hat{f}}_{n}^{\left(0\right)}=f_{0}>0$ for all *n*. Derivations of the list-mode MLEM algorithm are available in Levkovitz *et al* (2001) and Caucci (2012). Some stopping criteria can be found in Gaitanis *et al* (2010).

Critical to our treatment is the evaluation of $\text{pr}(\hat{A}|r,c)$. We followed an approach similar to the one delineated in Furenlid *et al* (2004) and Chen (2006), in which calibration data was collected and a Gaussian-shaped function was fit to the calibration events. Details can be found in appendix. With our approach, evaluation of $\text{pr}(\hat{A}|r,c)$ for fixed camera *c* and ***r*** only requires six coefficients.

System calibration for FastSPECT II entails moving a point-like radioactive source across the field of view to collect list-mode data from which estimate fitting coefficients (Chen 2006). As this process is often time-consuming, source decay must usually be taken into account. Moreover, one can only consider a finite set of positions at which collect calibration data. The alternative is to derive a complete analytical model for $\text{pr}(\hat{A}|r,c)$, but this would require precise knowledge of camera responses and their positions, as well as knowledge of position of all pinholes and their shapes. For this reason, we opted for estimating fitting coefficients for points ***r*** belonging to a 3D grid of points Γ, and whenever fitting coefficients at ***r*** not in Γ are needed, they are interpolated (as discussed in appendix), so that $\text{pr}(\hat{A}|r,c)$ can be evaluated quickly for any attribute vector $\hat{A}$ and point ***r*** and for any cam era *c*.

To summarize, we use photomultiplier tube signals to estimate the components of attribute vectors $\hat{A}$ with arbitrary precision. Our ML algorithm implemented on GPUs produces estimates $\hat{A}$ in real-time during the acquisition scan. Event attributes are collected into lists ${\hat{A}}^{\left(c\right)}=\left\{{\hat{A}}_{1}^{\left(c\right)},\ldots ,{\hat{A}}_{J_{c}}^{\left(c\right)}\right\}$ (one list for each camera *c* = 1, …, *C*), which are then used in the iterative expression in (23) to obtain estimates ${\hat{f}}^{\left(l\right)}$. By properly fitting measured calibration data, it is possible to evaluate $\text{pr}(\hat{A}|r,c)$ for any $\hat{A}$ and ***r*** and for all the cameras *c* = 1, …, *C*. By (19), this amounts to directly evaluate the kernel of the continuous-to-continuous operator L, which we use to obtain an estimate $\hat{f}$ to ***f*** that satisfies the forward model of (18).

## 6. Reconstruction results

### 6.1. Phantom studies

**Figure 3.**
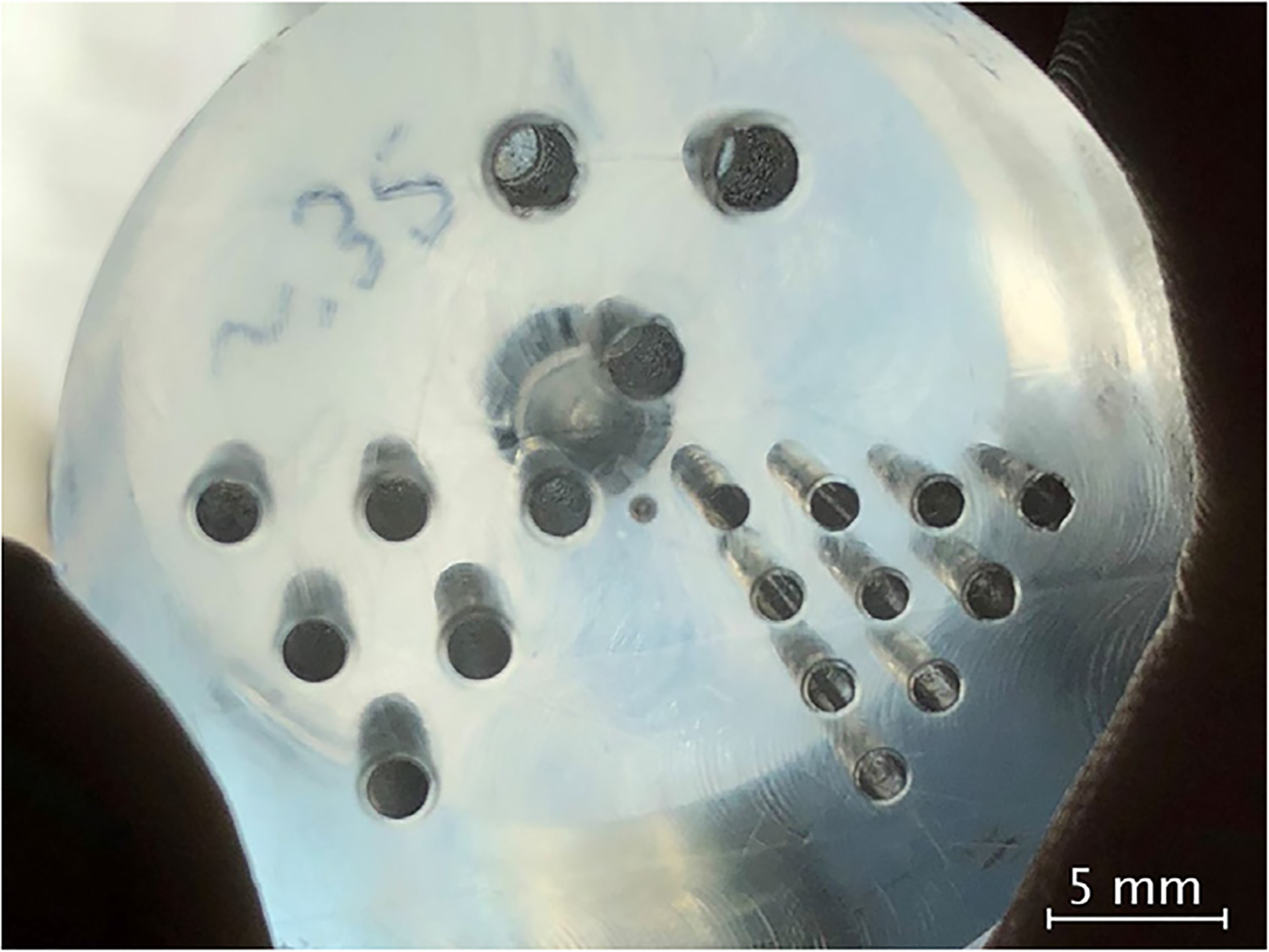
Optical image of the phantom used in the phantom studies.

In a first set of reconstructions, we imaged a Jaszczak-like phantom (shown in figure 3) whose bores were filled up with an aqueous solution of ^99m^Tc-based sodium pertechnetate. The metastable nuclear isomer ^99m^Tc decays to ^99^Tc with the emission of a gamma ray with a photon energy of 140 keV.

**Figure 4.**
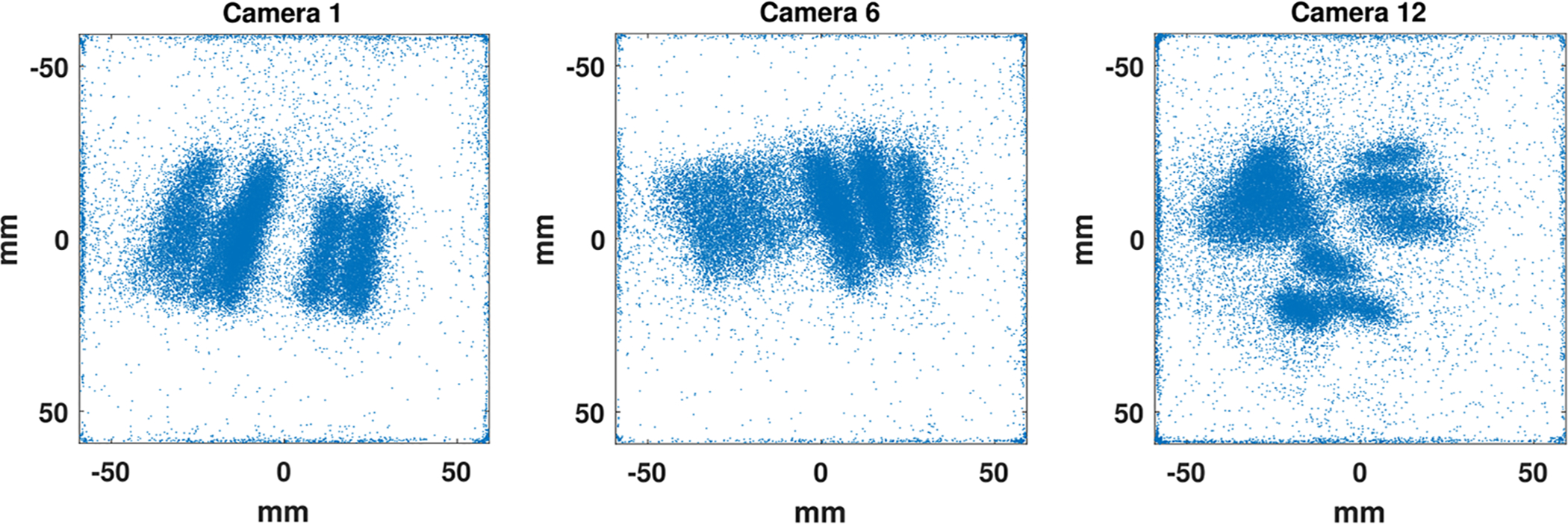
Scatter plots of raw estimated positions (after likelihood thresholding but before discarding estimates less than 4 mm away from any of the crystal edges) on the camera’s face obtained while imaging the phantom. Notice how some of the estimates cluster near the edges and corners of the crystal.

A total of about 13.55 · 10^6^ photons were collected while imaging the phantom with FastSPECT II. Of these, around 32.41% were discarded during likelihood thresholding (see section 5.2) or because estimated positions were less than 4 mm away from any of the crystal edges (see figure 4 for examples). This overall process left around 9.16 · 10^6^ counts available for reconstruction.

**Figure 5.**
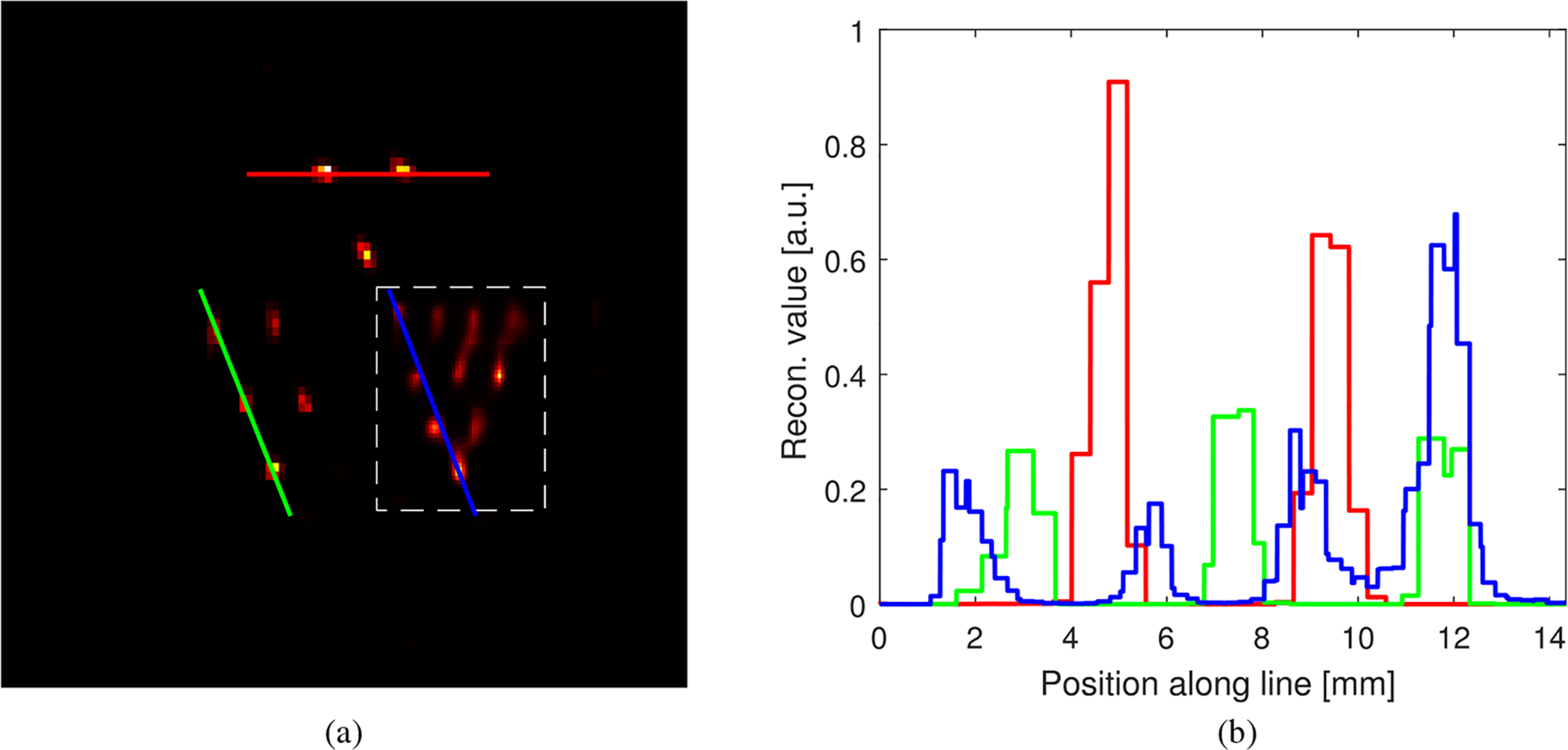
Phantom reconstruction results: (a) cross-section through the reconstructed object; (b) line profiles along selected lines.

List-mode data were reconstructed with the algorithm in (23) and at points ***r***_*n*_ arranged on a non-constant spacing grid. More specifically, we considered a coarse grid with a specific region of 2× finer spacing. Post-reconstruction nearest-neighbor resampling of the coarse regions was used to ease visualization without altering the process of image reconstruction. Figure 5(a) shows a cross-section of the reconstructed data in which the region of finer spacing was emphasized with a dashed box. (Notice that during preparation of the phantom, some of the bores were not properly filled with the radiotracer.) Some line profiles through the post-processed reconstructed data are shown as figure 5(b).

### 6.2. Reconstructions of KPC mouse

Imaging studies of FastSPECT II were performed in a genetically-modified K-ras^LSL.G12D/+^; p53^R172H/+^; PdxCre mouse model (Hingorani *et al* 2005, Westphalen and Olive 2012, Lee *et al* 2016) (often referred to as KPC mouse model) carrying spontaneous pancreatic ductal adenocarcinoma (PDAC). KPC mice develop a spectrum of premalignant lesions that ultimately progress over months to overt carcinoma with extensive stromal desmoplasia, similar to the most common morphology of PDAC observed in humans.

^99m^Tc-labeled 3P_4_-RGD_2_ dimer (^99m^Tc-3P_4_-RGD_2_), which binds to *α*_v_*β*_3_ integrin and indicates angiogenic activity in tumor stroma, was selected to image PDAC angiogenesis (Wang *et al* 2009). Integrin *α*_v_*β*_3_ is a cell-surface receptor with an exposed arginine-glycine-aspartate (RGD) binding site for a variety of extracellular matrix (ECM) proteins (Davis 1992).

**Figure 6.**
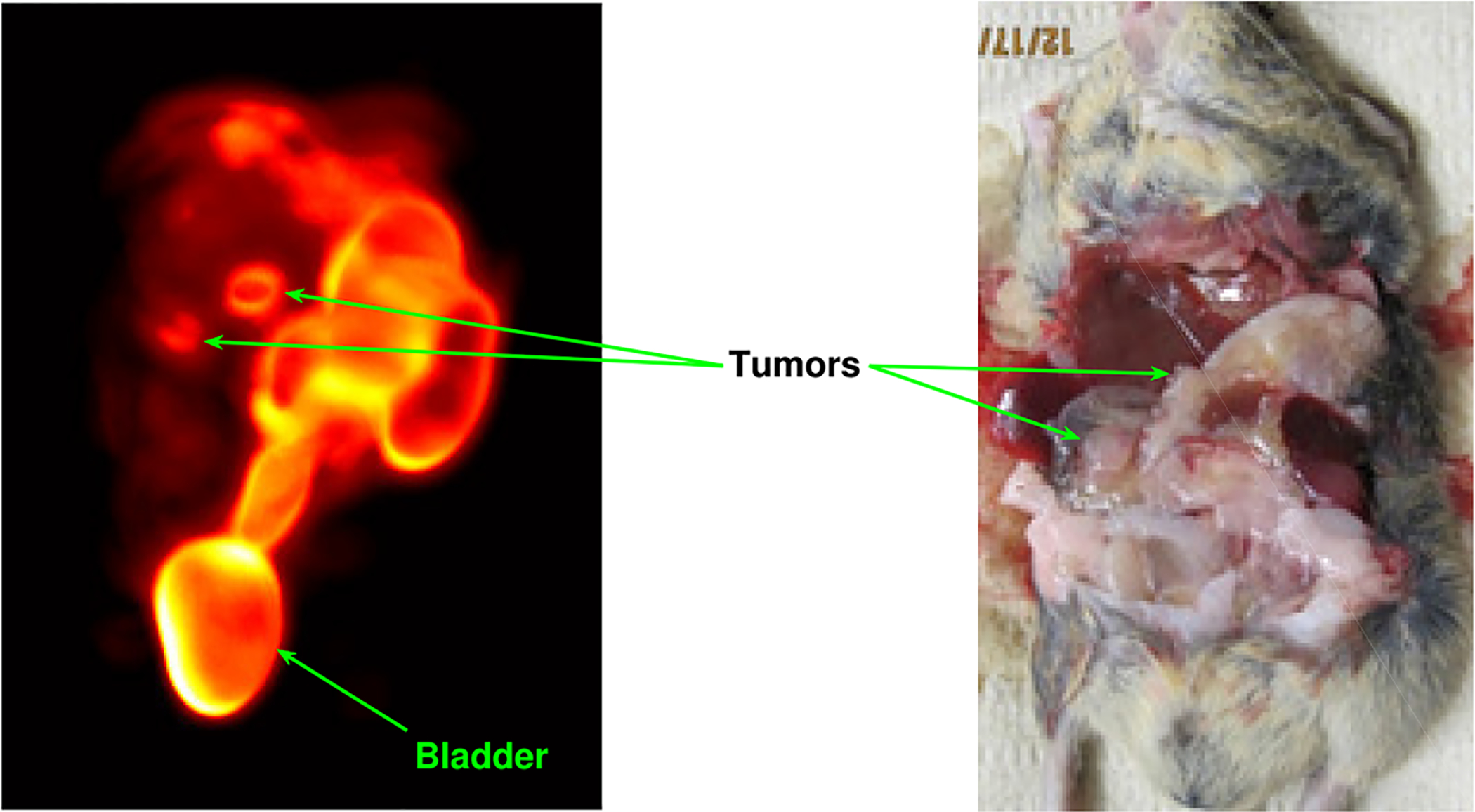
High-resolution FastSPECT II reconstructions compared with postmortem examination of a KPC mouse showing two lesions with high radioactive uptake in the pancreas.

Three hours after intravenous tracer injection (1.0 mCi of ^99m^Tc-3P_4_-RGD_2_) into the mouse, FastSPECT II imaging was performed. A total of 1.71 × 10^6^ events across the 16 cameras were collected, 34.21% of which were classified as scattered by likelihood thresholding and, therefore, rejected. This left about 1.13 × 10^6^ events available for reconstruction. High-resolution reconstructions (figure 6) showed two lesions with high radioactive uptake (arrows) in the upper right abdomen (left panel), which were consistent with the tumors observed in the mouse pancreas by postmortem examination (right panel).

All the reconstructions were obtained after 50 iterations of the method in (23). In other words, we assumed that the final reconstruction was ${\hat{f}}^{\left(50\right)}$. Renderings of ${\hat{f}}^{\left(50\right)}$ were obtained with AMIDE^5^.

To test the main point of this paper—that reconstruction methods based on continuous-to-continuous operators provide an advantage over algorithms that assume a discrete-to-discrete model for the imaging system—we considered and compared different sets of reconstructions in which:

- the number of iterations of the contracting-grid algorithm for the estimation of attribute vectors $\hat{A}$ was increased, thus allowing estimates $\hat{A}$ to take on values approximating a continuous domain; or,
- finer and finer system interpolation (see appendix A.2) was performed, thus allowing reconstructions on arbitrary sets of points (which are taken as approximations of continuous functions).

**Figure 7.**
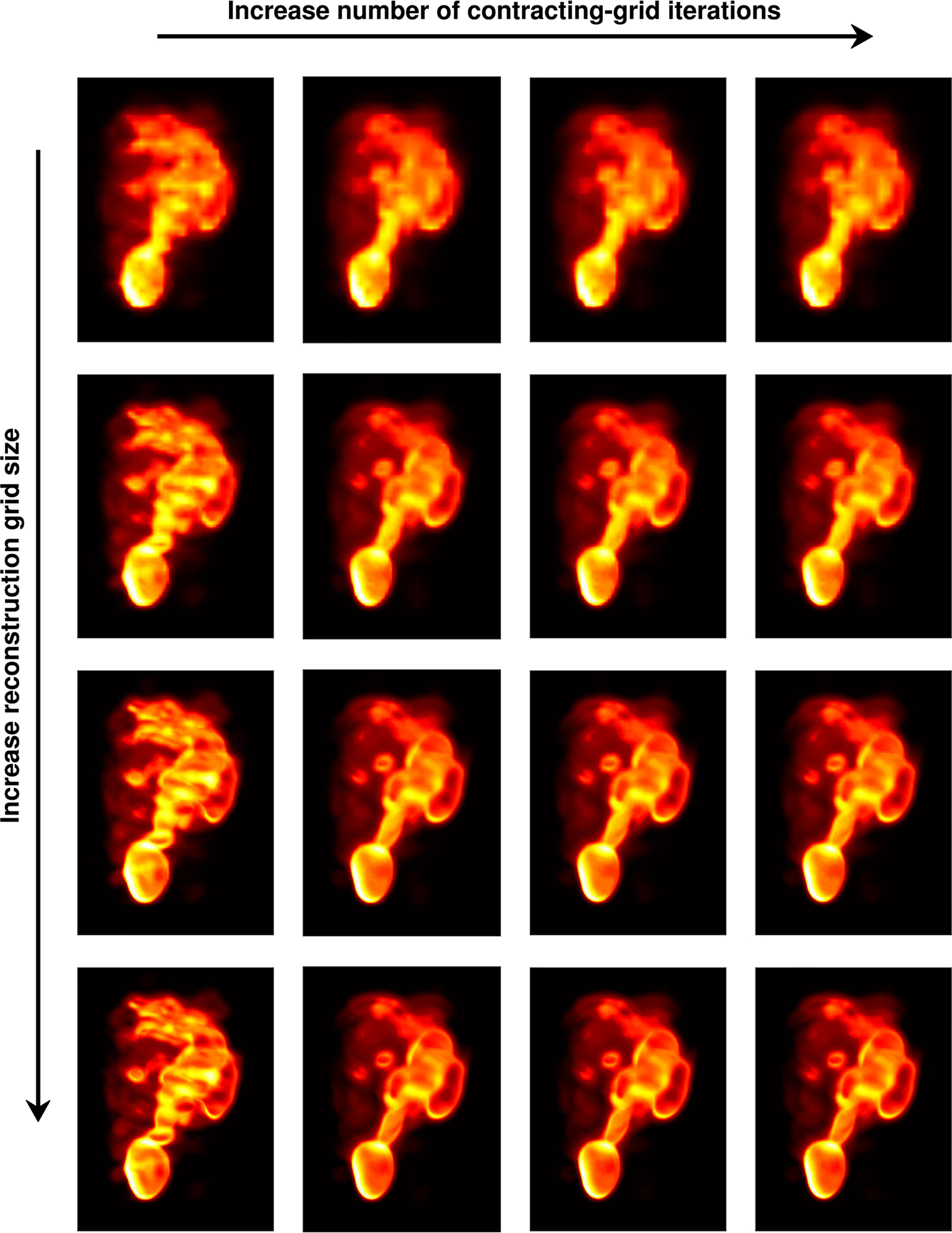
Reconstruction results in which the number of contracting-grid iterations was 4, 8, 12 and 16 (left to right) and the size of voxel arrays over which the data were reconstructed was 53 × 41 × 41, 105 × 81 × 81, 209 × 161 × 161 and 417 × 321 × 321 (top to bottom).

Reconstruction results are summarized in figure 7. In this figure, the number of contracting-grid iterations was varied from 4 (leftmost column) to 16 (rightmost column), and the reconstructions were obtained for voxel grids of four different sizes going from 53 × 41 × 41 (top row) to 417 × 321 × 321 (bottom row). Because of the nature of the contracting-grid algorithm itself (Caucci *et al* 2018), a linear increase in the number of iterations gives exponentially finer estimates of $\hat{A}$.

**Figure 8.**
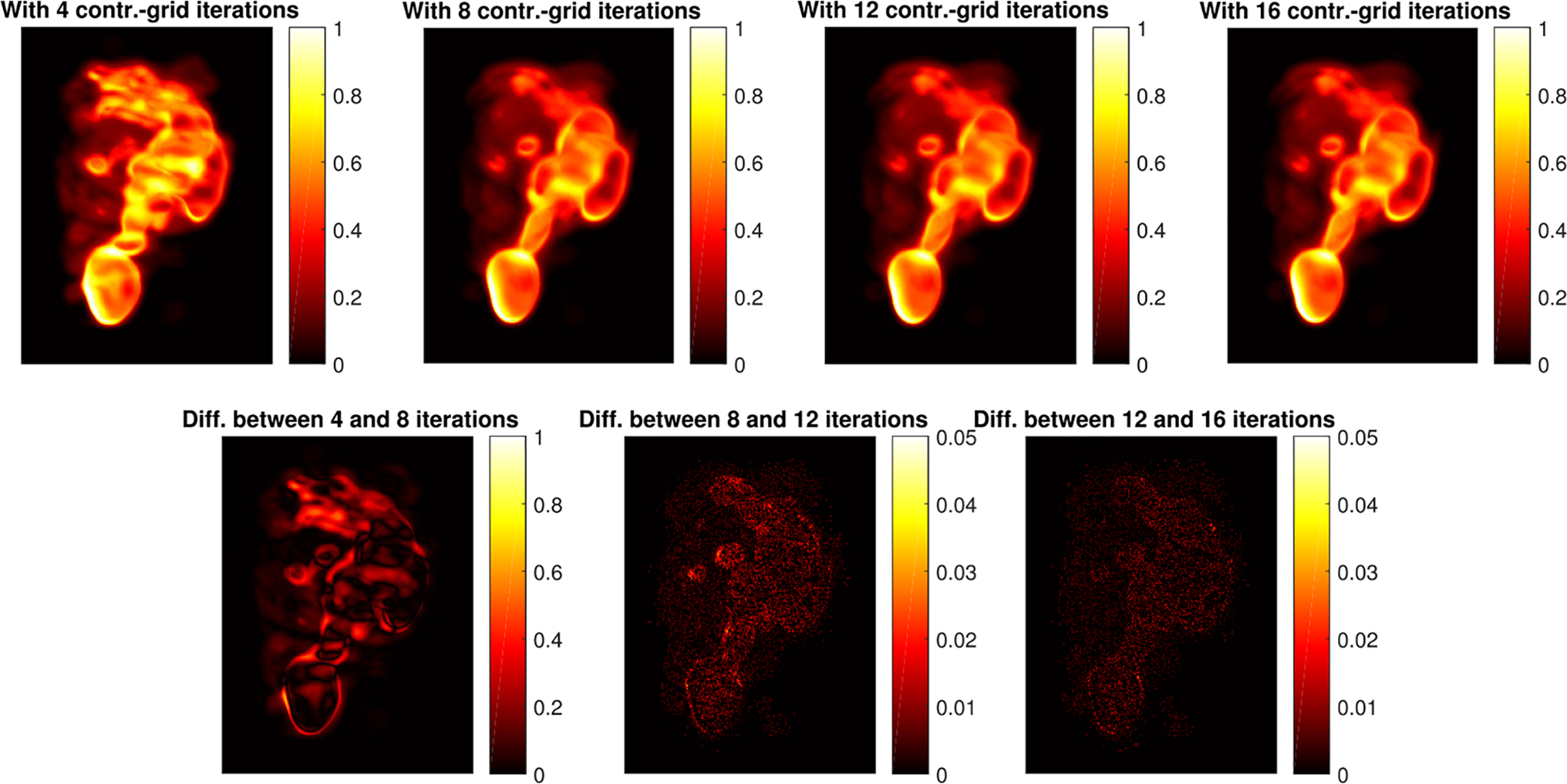
Reconstructions for a 417 × 321 × 321 voxel array and as the number of iterations in the contracting-grid search algorithm was varied over {4, 8, 12, 16}. Top row: reconstructed data; bottom row: differences between pairs of reconstructions.

Visual assessment of figure 7 shows that as the size of the voxel array used in the reconstruction increases, resolution improves and tumors are well resolved. Similarly, as the event estimates $\hat{A}=\left(\hat{X},\hat{Y}\right)$ are allowed to take value on a continuous domain, image resolution improves and the tumors (see left panel in figure 6) are better resolved. To better appreciate this fact, we report in the top row of figure 8 the reconstructions we obtained for a voxel grid of size 417 × 321 × 321 and different number of contracting-grid iterations. In the bottom row, we reported the absolute value of the difference between pairs of reconstructions, which were displayed as images. Notice the different color scales used in the images at the bottom of each figure.

We ran our codes on a server equipped with four Intel® Xeon® CPU E5–2698 processors, 256 GB of RAM and eight NVIDIA® Tesla® P100-SXM2 GPU accelerator cards. The software configuration included Linux openSUSE Leap 15.0, NVIDIA® CUDA SDK release 10.0, and GNU C compiler 7.3.1. Our codes heavily relied on GPU processing for the estimation of photon attributes (see section 5.2) and the calculation in (23). We designed our implementations to fully utilize all the GPU accelerators available. Estimation of photon attributes for all the 1.71 × 10^6^ events took less than half of a second, no matter how many iterations of the contracting-grid algorithm were performed. In fact, the algorithm just needs a few iterations to ‘localize’ an event to a small region of the search space. Subsequent iterations are much faster, as they rely on cached data for the evaluation of the MDRF fitting splines. On the other hand, reconstruction time is highly affected by the image-space granularity and the number of iterations during reconstruction. Table 1 reports running time for 50 iterations and different sizes of the voxel array. These results show that running time is proportional to the number of reconstruction points.

## 7. Future work

One possible follow-up research in this field might consider objective assessment of image quality (Barrett *et al* 1998) for continuous-to-continuous imaging systems. For example, a task-based figure-of-merit can be used to compare a continuous-to-continuous imaging system with an imaging system that use the traditional discrete-to-discrete model. In the case of a detection problem, such as detection of tumor necrosis, a meaningful figure-of-merit is the area under the receiver operating characteristic curve (Van Trees 1968). For an estimation problem, one could derive an expression for the list-mode Fisher information matrix (Fisher 1925), which also provides a lower bound on the variance any unbiased estimator (Van Trees 1968). Some preliminary work has been done in the context of estimating activity within a certain region of interest, and it was shown that continuous-to-continuous systems provided more accurate tracer uptake estimates compared to continuous-to-discrete systems (Jha and Frey 2015).

Although this paper has been limited to imaging with gamma-ray photons, the same methodology can be applied to imaging with charged particles, such as alpha and beta particles. Some work (Ding *et al* 2017b) has already been done in this direction, and it was shown that estimating additional parameters (such as direction and/or particle’s residual energy) besides positions, does provide enormous benefits in terms of reduced null space.

Other methods for reconstruction of a continuous functions have been introduced in the past decade. Among them, is the Backus–Gilbert method (Backus and Gilbert 1968, Kirsch *et al* 1988), originally introduced for the estimation of geological models from a finite set of noisy measurements. A more recent method (Jha *et al* 2015) for reconstruction of continuous functions uses the singular value decomposition of the imaging operator. A task-based comparison between these methods and the reconstruction approach developed in this paper will further shed some light on the advantages of image reconstruction algorithms based on continuous-to-continuous model.

## 8. Conclusions

We presented a new approach to image reconstruction for single-photon emission computed tomography (SPECT) that does not use a discrete-to-discrete representation of the imaging system. In fact, our approach models the imaging system via a continuous-to-continuous operator that maps the object (i.e. a function of continuous variables) to a function that takes as input an event attribute, which is assumed to take values on a continuous set. The reconstruction algorithm we propose is based on maximum-likelihood. It uses measured calibration data, and we showed how these calibration data can be interpolated to arbitrary precision.

We tested our approach on real data, which we collected with FastSPECT II. We used ^99m^Tc 3P_4_-RGD_2_ peptide to image a genetically-modified KPC mouse that developed pancreatic ductal adenocarcinoma in two different locations. By varying key estimation/interpolation parameters in our code, we were able to attain reconstructions on finer and finer grids, thus approximating reconstructions on a continuous domain. We did not perform a formal assessment of image quality. Instead, we relied on visual comparison of reconstructed volume renderings, which showed increased details as the limit of a continuous-to-continuous system was approached.

Our algorithms were implemented for a GPU architecture, which offers enormous parallel-computing capabilities as well as large amounts of fast memory. In particular, we took advantage of texture interpolation to rapidly retrieve calibration data. The algorithm we proposed to interpolate the system response avoids conditional branching, which are very time consuming on a GPU architecture. Moreover, our interpolation and reconstruction algorithm provides greater flexibility as they allow reconstructions on an arbitrary domain, not necessarily restricted to a uniform grid of points. Although GPUs were shown to be well suited for implementation of our parallel codes for maximum-likelihood estimation, other parallel architecture (e. g., field-programmable gate arrays, or FPGAs) might be considered as well.

## Figures and Tables

**Table 1.** Reconstruction time for different sizes of the voxel array.

Array size	Recon. time
53 × 41 × 41	39.52 s
105 × 81 × 81	289.40 s
209 × 161 × 161	37.66 min
417 × 321 × 321	298.25 min

**Figure A1.**
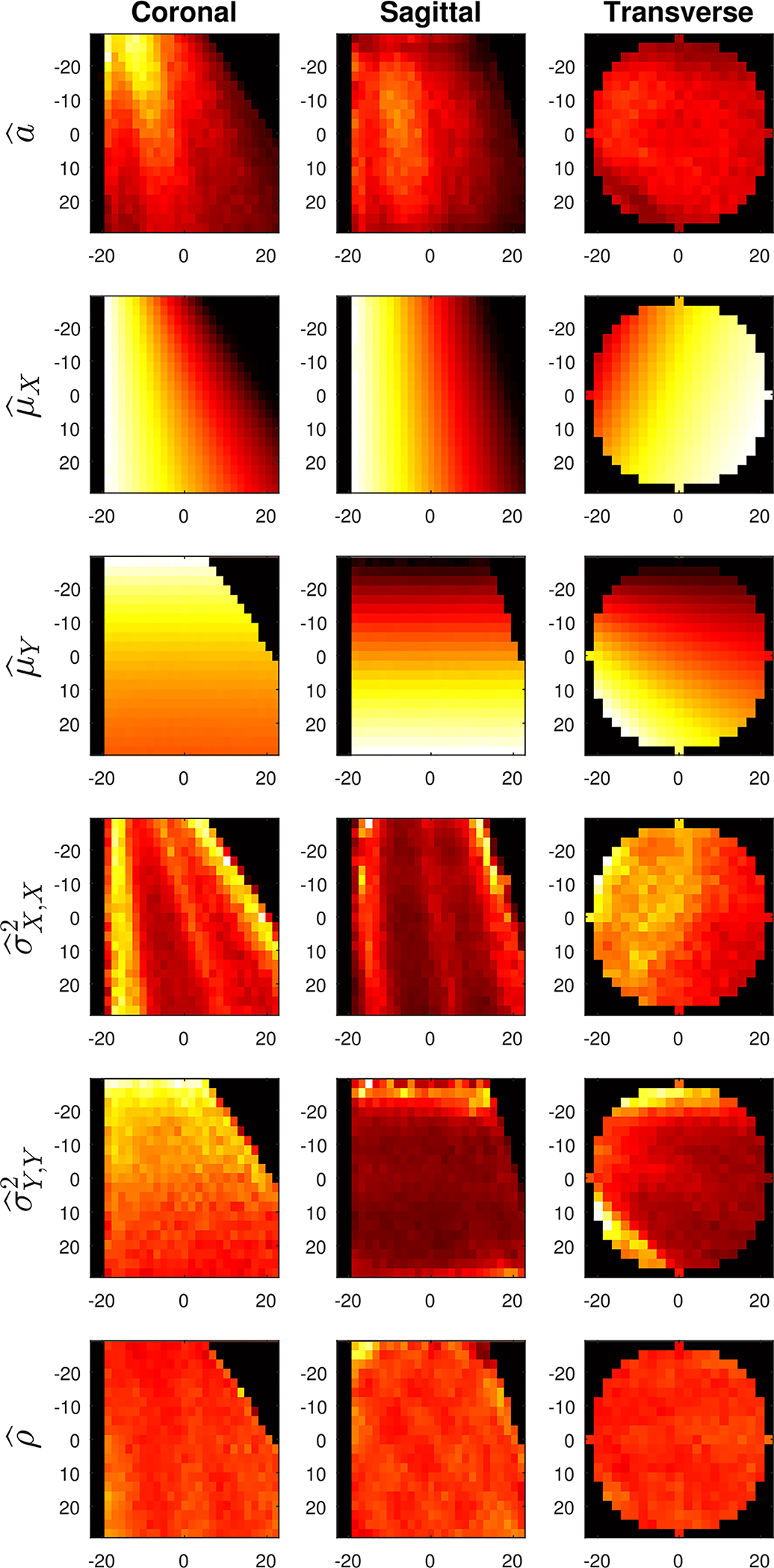
Plots of $\hat{a}\left(r_{n},c\right)$, ${\hat{\mu }}_{X}\left(r_{n},c\right)$, ${\hat{\mu }}_{Y}\left(r_{n},c\right)$, ${\hat{\sigma }}_{X,X}^{2}\left(r_{n},c\right)$, ${\hat{\sigma }}_{Y,Y}^{2}\left(r_{n},c\right)$ and $\hat{\rho }\left(r_{n},c\right)$ for one of the cameras and calculated according to (A.4)–(A.11). These plots show that estimated coefficients vary smoothly as the point ***r***_***n***_ is moved over the field of view (position along the axes are in units of millimeters).

**Figure A2.**
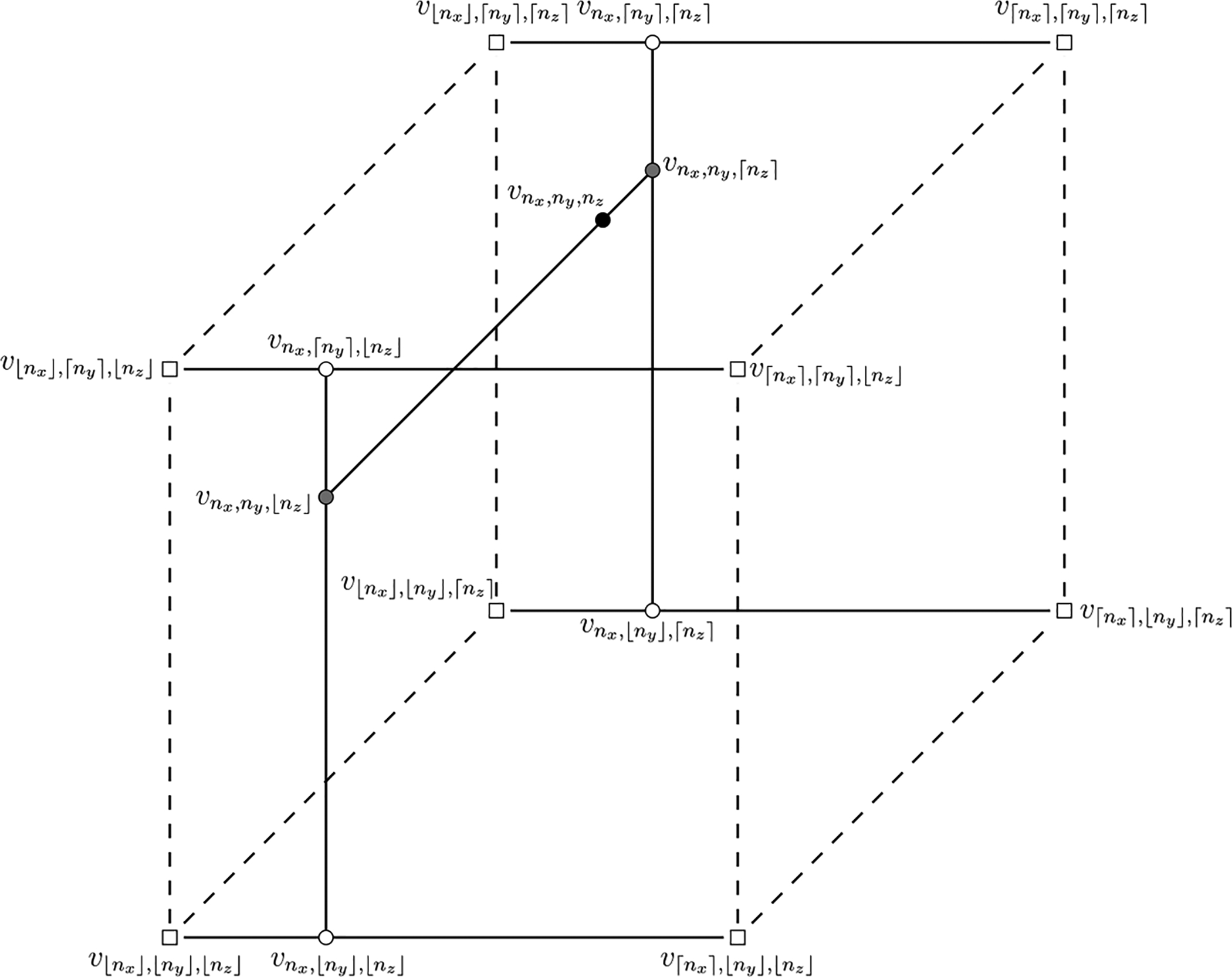
Interpolation of $v_{n_{x},n_{y},n_{z}}$.

## Footnotes

5 http://amide.sourceforge.net/index.html

## Appendix. Estimation of system response

In our implementation, probabilities $\text{pr}(\hat{A}|r,c)$ for $\hat{A}=\left(\hat{X},\hat{Y}\right)$ were approximated by a 2D Gaussian function of the form:

(A.1) $$p(\hat{X},\hat{Y};r,c)=\hat{a}(r,c)$$

(A.2) $$\times \text{exp}\left\{\frac{\hat{\rho }(r,c)u_{X}u_{Y}-\left[u_{X}^{2}+u_{Y}^{2}\right]/2}{1-{\left[\hat{\rho }(r,c)\right]}^{2}}\right\},$$

in which we defined

(A.3) $$u_{X}=\frac{\hat{X}-{\hat{\mu }}_{X}(r,c)}{\sqrt{{\hat{\sigma }}_{X,X}^{2}(r,c)}},\;u_{Y}=\frac{\hat{Y}-{\hat{\mu }}_{Y}(r,c)}{\sqrt{{\hat{\sigma }}_{Y,Y}^{2}(r,c)}},$$

and $\hat{a}(r,c)$, ${\hat{\mu }}_{X}(r,c)$, ${\hat{\mu }}_{Y}(r,c)$, ${\hat{\sigma }}_{X,X}^{2}(r,c)$, ${\hat{\sigma }}_{Y,Y}^{2}(r,c)$ and $\hat{\rho }(r,c)$ are fitting coefficients. Calculation of these fitting coefficients at any point ***r*** and for any camera *c* requires two separate steps:

- system calibration to estimate fitting coefficients for ***r*** belonging to a uniform grid;
- interpolation of the fitting coefficients obtained in the previous step.

The Gaussian model of (A.1) was chosen for its simplicity. Depending on the system geometry, other choices are possible. This includes sums of Gaussian functions (for the case of a multi-pinhole imaging system), or a more general model for $p(\hat{X},\hat{Y};r,c)$ in (A.1) based on, fore example, the 2D Pearson type VII distribution (Kotz 1975).

### A.1. System calibration

In a typical system calibration procedure, a point source is moved across the field of view by means of computer-controlled motorized stages. The position of the point source will be denoted as ***r***_***n***_, in which we defined ***n*** = (*n*_*x*_, *n*_*y*_, *n*_*z*_) for *n*_*x*_ = 1, …,*N*_*x*_, *n*_*y*_ = 1, …, *N*_*y*_ and *n*_*z*_ = 1, …, *N*_*z*_. Points ***r***_***n***_ are arranged on a uniform grid, which we will denote as Γ, and adjacent points are separated by a known distance *δ*.

During calibration, list-mode data ${\hat{A}}_{n}^{\left(c\right)}$ are collected at each of the *N*_*x*_*N*_*y*_*N*_*z*_ points positions ***r***_***n***_ ∈ Γ and for each of the *C* cameras. We will assume that ${\hat{A}}_{n}^{\left(c\right)}$ contains $J_{n}^{\left(c\right)}$ attribute vectors ${\hat{A}}_{j}=\left({\hat{X}}_{j},{\hat{Y}}_{j}\right)$, in which $j=1,\ldots ,J_{n}^{\left(c\right)}$. Notice that ${\hat{A}}_{j}$ (and, consequently, ${\hat{X}}_{j}$ and ${\hat{Y}}_{j}$) do depend on ***n*** and *c*, but we have discarded these dependencies for notational convenience. We used our GPU-based maximum-likelihood code to estimate attribute vectors ${\hat{A}}_{j}$ according to (20). From ${\hat{A}}_{1},\ldots ,{\hat{A}}_{J_{n}^{\left(c\right)}}$, Gaussian fitting coefficients for $p\left(\hat{X},\hat{Y};r_{n},c\right)$ as in (A.1) are calculated as:

(A.4) $${\hat{\mu }}_{X}\left(r_{n},c\right)=\frac{1}{J_{n}^{\left(c\right)}}\sum_{j=1}^{J_{n}^{\left(c\right)}}{\hat{X}}_{j},$$

(A.5) $${\hat{\mu }}_{Y}\left(r_{n},c\right)=\frac{1}{J_{n}^{\left(c\right)}}\sum_{j=1}^{J_{n}^{\left(c\right)}}{\hat{Y}}_{j},$$

(A.6) $${\hat{\sigma }}_{X,X}^{2}\left(r_{n},c\right)=\frac{1}{J_{n}^{\left(c\right)}-1}\sum_{j=1}^{J_{n}^{\left(c\right)}}{\left[{\hat{X}}_{j}-{\hat{\mu }}_{X}\left(r_{n},c\right)\right]}^{2},$$

(A.7) $${\hat{\sigma }}_{Y,Y}^{2}\left(r_{n},c\right)=\frac{1}{J_{n}^{\left(c\right)}-1}\sum_{j=1}^{J_{n}^{\left(c\right)}}{\left[{\hat{Y}}_{j}-{\hat{\mu }}_{Y}\left(r_{n},c\right)\right]}^{2},$$

(A.8) $${\hat{\sigma }}_{X,Y}^{2}\left(r_{n},c\right)={\hat{\sigma }}_{Y,X}^{2}\left(r_{n},c\right)=\frac{1}{J_{n}^{\left(c\right)}-1}$$

(A.9) $$\times \sum_{j=1}^{J_{n}^{\left(c\right)}}\left[{\hat{X}}_{j}-{\hat{\mu }}_{X}\left(r_{n},c\right)\right]\left[{\hat{Y}}_{j}-{\hat{\mu }}_{Y}\left(r_{n},c\right)\right],$$

(A.10) $$\hat{\rho }\left(r_{n},c\right)=\frac{{\hat{\sigma }}_{X,Y}^{2}\left(r_{n},c\right)}{\sqrt{{\hat{\sigma }}_{X,X}^{2}\left(r_{n},c\right){\hat{\sigma }}_{Y,Y}^{2}\left(r_{n},c\right)}},$$

(A.11) $$\hat{a}\left(r_{n},c\right)=\frac{J_{n}^{\left(c\right)}}{2\pi \sqrt{{\hat{\sigma }}_{X,X}^{2}\left(r_{n},c\right){\hat{\sigma }}_{Y,Y}^{2}\left(r_{n},c\right)}\sqrt{1-{\left[\hat{\rho }\left(r_{n},c\right)\right]}^{2}}}.$$

The expression for ${\hat{\mu }}_{X}\left(r_{n},c\right)$, ${\hat{\mu }}_{Y}\left(r_{n},c\right)$, ${\hat{\sigma }}_{X,X}^{2}\left(r_{n},c\right)$, ${\hat{\sigma }}_{Y,Y}^{2}\left(r_{n},c\right)$ and ${\hat{\sigma }}_{X,Y}^{2}\left(r_{n},c\right)$ above are unbiased estimator for the corresponding unknown quantities.

### A.2. Interpolation

The system calibration process outlined above is often performed on a coarse grid Γ of points ***r***_***n***_. Time constraints (due to radioactive decay in the point source used for calibration) along with the necessity of collecting a few thousand events at each point ***r***_***n***_ limit the separation *δ* between two adjacent points to around 2 mm (Chen 2006). With a typical short-lived isotope used in medical imaging (e.g. ^99m^Tc), collecting enough calibration data at each point of a finer grid while covering the entire field of view (see figure 2) is infeasible. For this reason, we have developed a method for fast calculation of the fitting coefficients ${\hat{\mu }}_{X}\left(r,c\right)$, ${\hat{\mu }}_{Y}\left(r,c\right)$, ${\hat{\sigma }}_{X,X}^{2}\left(r,c\right)$, ${\hat{\sigma }}_{Y,Y}^{2}\left(r,c\right)$ and ${\hat{\sigma }}_{X,Y}^{2}\left(r,c\right)$ any point ***r*** and for any camera *c* from quantities estimated in (A.4)–(A.11). Figure A1 reports sample plots of $\hat{a}\left(r_{n},c\right)$, ${\hat{\mu }}_{X}\left(r_{n},c\right)$, ${\hat{\mu }}_{Y}\left(r_{n},c\right)$, ${\hat{\sigma }}_{X,X}^{2}\left(r_{n},c\right)$, ${\hat{\sigma }}_{Y,Y}^{2}\left(r_{n},c\right)$ and $\hat{\rho }\left(r_{n},c\right)$ as estimated according to (A.4)–(A.11) and along perpendicular planes intersection at the center of the field of view.

The first step in our method is to diagonalize the following matrix:

(A.12) $$K\left(r_{n},c\right)=\left(\begin{array}{cc} {\hat{\sigma }}_{X,X}^{2}\left(r_{n},c\right) & {\hat{\sigma }}_{X,Y}^{2}\left(r_{n},c\right)\\ {\hat{\sigma }}_{X,Y}^{2}\left(r_{n},c\right) & {\hat{\sigma }}_{Y,Y}^{2}\left(r_{n},c\right) \end{array}\right)$$

so that:

(A.13) $$K\left(r_{n},c\right)=R_{\phi \left(r_{n},c\right)}\Lambda R_{\phi \left(r_{n},c\right)}^{-1},$$

where

(A.14) $$R_{\phi \left(r_{n},c\right)}=\left(\begin{array}{cc} \text{cos }\phi \left(r_{n},c\right) & -\text{sin }\phi \left(r_{n},c\right)\\ \text{sin }\phi \left(r_{n},c\right) & \text{cos }\phi \left(r_{n},c\right) \end{array}\right)$$

and

(A.15) $$\Lambda =\left(\begin{array}{cc} \lambda_{X}\left(r_{n},c\right) & 0\\ 0 & \lambda_{Y}\left(r_{n},c\right) \end{array}\right).$$

Quantities *ϕ*(***r***_***n***_, *c*), *λ*_*X*_(***r***_***n***_, *c*) and *λ*_*Y*_ (***r***_***n***_, *c*) are calculated as follows:

(A.16) $$\phi \left(r_{n},c\right)=\frac{1}{2}\text{arctan}\left(\frac{2{\hat{\sigma }}_{X,Y}^{2}\left(r_{n},c\right)}{{\hat{\sigma }}_{X,X}^{2}\left(r_{n},c\right)-{\hat{\sigma }}_{Y,Y}^{2}\left(r_{n},c\right)}\right),$$

(A.17) $$\lambda_{X}\left(r_{n},c\right)=\frac{1}{2}\left[{\hat{\sigma }}_{X,X}^{2}\left(r_{n},c\right)+{\hat{\sigma }}_{Y,Y}^{2}\left(r_{n},c\right)+\Delta \right],$$

(A.18) $$\lambda_{Y}\left(r_{n},c\right)=\frac{1}{2}\left[{\hat{\sigma }}_{X,X}^{2}\left(r_{n},c\right)+{\hat{\sigma }}_{Y,Y}^{2}\left(r_{n},c\right)-\Delta \right],$$

in which,

(A.19) $$\Delta ={\left\{{\left[{\hat{\sigma }}_{X,X}^{2}\left(r_{n},c\right)-{\hat{\sigma }}_{Y,Y}^{2}\left(r_{n},c\right)\right]}^{2}+4{\left[\hat{\rho }\left(r_{n},c\right){\hat{\sigma }}_{X,X}\left(r_{n},c\right){\hat{\sigma }}_{Y,Y}\left(r_{n},c\right)\right]}^{2}\right\}}^{1/2}.$$

Interpolation was performed according to the diagram show in figure A2, in which we used the placeholder ‘*v*’ to denote one among $\hat{a}$, ${\hat{\mu }}_{X}$, ${\hat{\mu }}_{Y}$, *ϕ*, *λ*_*X*_ or *λ*_*Y*_.

For simplicity, we assumed that any interpolated value was indexed by a subscript vector (*n*_*x*_, *n*_*y*_, *n*_*z*_) in which at least one in *n*_*x*_, *n*_*y*_ or *n*_*z*_ is non-integer. In figure A2 we used the ‘floor’ (⌊…⌋) and ‘ceiling’ (⌈…⌉) notation to calculate subscripts for the non-interpolated values of *v* that ‘surrounds’ $v_{n_{x},n_{y},n_{z}}$ and are used in the interpolation. These non-interpolated values, which by construction corresponds to points in Γ, are marked in figure A2 with squares. Weighted two-value average was used to interpolate two values at a time as follows:

(A.20) $$v_{n_{x},\left\lfloor n_{y}\right\rfloor ,\left\lfloor n_{z}\right\rfloor }=W\left(v_{\left\lfloor n_{x}\right\rfloor ,\left\lfloor n_{y}\right\rfloor ,\left\lfloor n_{z}\right\rfloor },v_{\left\lceil n_{x}\right\rceil ,\left\lfloor n_{y}\right\rfloor ,\left\lfloor n_{z}\right\rfloor },n_{x}-\left\lfloor n_{x}\right\rfloor \right),\;v_{n_{x},\left\lceil n_{y}\right\rceil ,\left\lfloor n_{z}\right\rfloor }=W\left(v_{\left\lfloor n_{x}\right\rfloor ,\left\lceil n_{y}\right\rceil ,\left\lfloor n_{z}\right\rfloor },v_{\left\lceil n_{x}\right\rceil ,\left\lceil n_{y}\right\rceil ,\left\lfloor n_{z}\right\rfloor },n_{x}-\left\lfloor n_{x}\right\rfloor \right),\;v_{n_{x},\left\lfloor n_{y}\right\rfloor ,\left\lceil n_{z}\right\rceil }=W\left(v_{\left\lfloor n_{x}\right\rfloor ,\left\lfloor n_{y}\right\rfloor ,\left\lceil n_{z}\right\rceil },v_{\left\lceil n_{x}\right\rceil ,\left\lfloor n_{y}\right\rfloor ,\left\lceil n_{z}\right\rceil },n_{x}-\left\lfloor n_{x}\right\rfloor \right),\;v_{n_{x},\left\lceil n_{y}\right\rceil ,\left\lceil n_{z}\right\rceil }=W\left(v_{\left\lfloor n_{x}\right\rfloor ,\left\lceil n_{y}\right\rceil ,\left\lceil n_{z}\right\rceil },v_{\left\lceil n_{x}\right\rceil ,\left\lceil n_{y}\right\rceil ,\left\lceil n_{z}\right\rceil },n_{x}-\left\lfloor n_{x}\right\rfloor \right),\;v_{n_{x},n_{y},\left\lfloor n_{z}\right\rfloor }=W\left(v_{n_{x},\left\lfloor n_{y}\right\rfloor ,\left\lfloor n_{z}\right\rfloor },v_{n_{x},\left\lceil n_{y}\right\rceil ,\left\lfloor n_{z}\right\rfloor },n_{y}-\left\lfloor n_{y}\right\rfloor \right),\;v_{n_{x},n_{y},\left\lceil n_{z}\right\rceil }=W\left(v_{n_{x},\left\lfloor n_{y}\right\rfloor ,\left\lceil n_{z}\right\rceil },v_{n_{x},\left\lceil n_{y}\right\rceil ,\left\lceil n_{z}\right\rceil },n_{y}-\left\lfloor n_{y}\right\rfloor \right),\;v_{n_{x},n_{y},n_{z}}=W\left(v_{n_{x},n_{y},\left\lfloor n_{z}\right\rfloor },v_{n_{x},n_{y},\left\lceil n_{z}\right\rceil },n_{z}-\left\lfloor n_{z}\right\rfloor \right),$$

in which *W*(*v*_1_, *v*_2_, *γ*) is the weighting two-value average function defined as:

(A.21) $$W\left(v_{1},v_{2},\gamma \right)=\{\begin{array}{cc} {\left[(1-\gamma )v_{1}^{-1}+\gamma v_{2}^{-1}\right]}^{-1} & \text{if }v=\lambda_{X}\text{ or }v=\lambda_{Y},\\ (1-\gamma )v_{1}+\gamma v_{2} & \text{otherwise}. \end{array}$$

In other words, *W*(*v*_1_, *v*_2_, *γ*) is the two-value weighted arithmetic mean between *v*_1_ and *v*_2_ (with weights 1 – *γ* and *γ*, respectively) if $v=\hat{a}$, $v={\hat{\mu }}_{X}$, $v={\hat{\mu }}_{Y}$ or *v* = *ϕ*; while *W*(*v*_1_, *v*_2_, *γ*) is the two-value weighted harmonic *v* = *λ*_*X*_ or *v* = *γ*_*Y*_.

Notice that the scheme in (A.20) operates on one dimension at a time; this is emphasized in figure A2 by solid lines connecting the pairs of points used in (A.20). Furthermore, for any integer number *n*, we have ⌊*n*⌋ = ⌈*n*⌉ = *n*, so that the scheme in (A.20) along with the definition of *W*(*v*_1_, *v*_2_, *γ*) in (A.21) effectively performs no interpolation along the dimension(s) for which the corresponding index(es) in (*n*_*x*_, *n*_*y*_, *n*_*z*_) are integer. Although this does perform unnecessary computation when one of more indeces in (*n*_*x*_, *n*_*y*_, *n*_*z*_) are integer, in practice, it is well suited for parallel implementation due to the absence of conditional branching.
